# Establishment of a quantitative *in vivo* method for estimating adipose tissue volumes and the effects of dietary soy sauce oil on adipogenesis in medaka, *Oryzias latipes*

**DOI:** 10.1371/journal.pone.0205888

**Published:** 2018-10-18

**Authors:** Yasuhiro Tonoyama, Masaki Tsukada, Yoshimasa Imai, Matoki Sanada, Syota Aota, Gouhei Oka, Shozo Sugiura, Nobuaki Hori, Hiroyuki Kawachi, Yoshiko Shimizu, Nobuyoshi Shimizu

**Affiliations:** 1 Graduate School of Bioscience, Nagahama Institute for Bioscience and Technology, Nagahama, Shiga, Japan; 2 Division of admission Center, Nagahama Institute for Bioscience and Technology, Nagahama, Shiga, Japan; 3 School of Environmental Sciences, The University of Shiga Prefecture, Hikone, Shiga, Japan; 4 Division of Research Management and External Cooperation, Nagahama Institute for Bioscience and Technology, Nagahama, Shiga, Japan; 5 Faculty of Health Sciences, Kyorin University, Mitaka, Tokyo, Japan; Universitat Politècnica de València, SPAIN

## Abstract

Adipose tissue, which is conserved in higher eukaryotes, plays central roles in controlling the body’s energy balance, including excess energy storage and energy expenditure during starvation. In adipogenesis, intranuclear receptor, peroxisome proliferator–activated receptor gamma (PPARγ) is a key molecule, and PPARγ agonists can promote adipogenesis. Many studies on the *in vitro* screening of PPARγ agonists with compounds derived from various materials have been reported; however, *in vivo* assays for quick examination of these feeding effects have not been established. In this study, we developed a technique using a lipophilic fluorescent reagent, Nile red to quantitatively estimate the adipose tissue volumes by using Japanese rice fish, medaka (*Oryzias latipes*) and studied effects of dietary soy sauce oil (SSO), which is a discarded by-product from Japanese traditional food and is known to have PPARγ-agonistic activity, on adipogenesis. We found that SSO feeding increased the adipose tissue volumes, and the expression levels of adipogenesis-related genes increased in these medaka larvae. These results suggest that SSO feeding increases the adipose tissue volumes through adipogenesis promotion by PPARγ-agonistic activity in medaka, and medaka is a powerful model for studying adipogenesis. Furthermore, our study also demonstrates the availability of SSO as a dietary additive for farmed fish.

## Introduction

Adipose tissue, which is conserved in higher eukaryotes, plays central roles in controlling the body’s energy balance, including excess energy storage and energy expenditure during starvation. It is classified into two types: white adipose tissue (WAT) and brown adipose tissue (BAT). WAT stores excess energy as triglycerides, whereas BAT contributes to thermogenic activity [1, 2]. Obesity is a condition in which excess WAT accumulates in the visceral and subcutaneous tissues of the body. It is a risk factor for serious diseases such as diabetes mellitus, cardiovascular disease, and metabolic syndrome [3, 4]. On the other hand, farmed animals like beef cattle, pigs, and fish that have high-quality fat in their bodies are traded at high prices, and people try to increase the adipose tissue content in meat by various approaches [5–8]. Therefore, knowledge of adipose tissues and the factors affecting adipogenesis is beneficial for not only human health care but also aquaculture and the livestock industry.

During adipogenesis, the number of adipocytes increases because of preadipocyte proliferation and because of the clonal expansion that occurs at an early stage of differentiation. Subsequently, adipocytes enlarge through the intake of free fatty acids and lipogenesis promotion [9–12]. In these processes, peroxisome proliferator-activated receptor gamma (PPARγ), intranuclear transcription factor, forms a complex with the retinoid X receptor (RXR) and its ligands [13, 14]. The complex binds to the PPAR response element (PPRE) in genomic DNA and induces the expression of adipocyte-related genes including *aP2*, *adiponectin*, and *ACVR1C* [15,16], resulting in formation of the adipose tissue. Notably, *aP2* and *adiponectin* are the target genes of PPARγ [16,17]. Following secretion from the adipose tissues, the adipokine, adiponectin increases insulin sensitivity in various target tissues [18]. Adipocyte protein 2 (aP2) is also known as fatty acid-binding protein 4 (FABP4) and acts as a carrier protein for fatty acids in intracellular lipid metabolism. Activin receptor type 1C (ACVR1C, also known ALK7) is a type I receptor of the TGFβ family and is regarded as a final differentiation marker of adipogenesis, reflecting its roles in fat accumulation in the adipocytes [19,20]. Therefore, PPARγ is a key molecule in adipogenesis, and dietary PPARγ agonists can promote adipogenesis *in vivo*.

Several studies screening PPARγ agonists among compounds derived from various materials have been reported [21–23]. Kawachi et al. screened PPARγ agonists using *in vitro* luciferase assays of by-products that are generated during manufacturing of fermented foods, and then showed that soy sauce oil (SSO) has PPARγ-agonistic activity [24]. Soy sauce is a traditional Japanese fermented food that is made from soybeans, and SSO is a by-product of soy sauce production and is produced in a large amount [25]. Hence, the effective doses of dietary SSO may promote adipogenesis safely *in vivo*, and may ameliorate economic and environmental concerns relating to nutrition [26, 27]. However, *in vivo* assays for rapid and convenient examinations of these feeding effects on adipogenesis have not been established.

The Japanese rice fish, medaka (*Oryzias latipes*) is a powerful model to study organogenesis in vertebrates because (i) it is a vertebrate and has various organs similar to humans; (ii) it breeds readily and grows into an adult in a relatively short period (2–3 months); (iii) there are abundant inbred lines; (iv) researchers can use accurate genome information; and (v) the fish’s transparent skin, especially at the larval stage, enables us to easily observe some organs and tissues, such as adipose tissue, blood vessels, and bones [28–30]. These advantages can facilitate effective feeding tests, drug screening and genetic studies, in which many genetically homogenous organisms need to be collected. Moreover, medaka also have adipose tissue in which adipogenesis-related orthologues are expressed [31], suggesting that mechanisms of adipogenesis are also conserved in the medaka. Therefore, these facts suggest that medaka can be a useful model to study adipogenesis in vertebrates and to screen for compounds that can affect the processes.

In this study, we built an experimental system using medaka to estimate adipose tissue volumes quantitatively and showed that SSO feeding can promote adipogenesis in medaka. Our results demonstrate that the screening system using medaka is a powerful approach to assessing effects of various materials on adipogenesis *in vivo*, and also the availability of SSO as a dietary additive for farmed fish.

## Materials and methods

### Fish maintenance

All procedures were approved by the Animal Care and Use Committee of Nagahama Institute for Bioscience and Technology. Fish from the inbred Cab strain of the medaka, sourced from the Carolina Biological Supply (NC, USA), were supplied by NBRP Medaka (http://shigen.nig.ac.jp/medaka/). They were maintained under an artificial photoperiod of 14 hours light: 10 hours darkness (14L:10D) at an ambient temperature of 28°C. Embryos were produced by natural mating and separated according to their morphology, as previously described by Iwamatsu [32].

### Luciferase assay for PPARγ ligand screening

Monkey CV1 kidney cells (ATCC, VA, USA) were cultured at 37°C in a humidified atmosphere of 5% CO_2_ in Dulbecco’s modified Eagle’s medium (DMEM) supplemented with 5% fetal bovine serum (FBS). Luciferase assays were conducted using a *GAL4*/*PPARγ* chimera assay system [22]. We transfected pM-*hPPARγ* (an expression plasmid for a chimeric protein for the *GAL4* DNA-binding domain [*GAL4BD*] and the human *PPARγ* [*hPPARγ*] ligand-binding domain), p4xUASg-tk-luc (a reporter plasmid), and pSV-β-galactosidase (an internal control plasmid for normalizing transfection efficiency) into CV1 cells using Lipofectamine (Invitrogen, CA, USA), according to the manufacturer’s instructions.

Transfected cells were seeded in 96-well plates and incubated for 24 h in a medium containing 1–100 μg/mL of soybean oil (SBO), soy sauce oil (SSO) or a control vehicle (0.1% dimethyl sulfoxide, DMSO). Luciferase activity was assayed using the Steady-Glo Luciferase Assay System (Promega, WI, USA), according to the manufacturer’s protocol, in a Centro XS^3^ LB 960 fluorescence microplate reader (Berthold, Bad Wildbad, Germany). All CV1 cells that were transiently transfected were assayed for β-galactosidase (β-gal) activity by adding 180 μL β-gal assay buffer (0.1 M PBS, pH 7.2, 2.8 mM MgCl_2_, 133 mM 2-mercaptoethanol, 2.8 mg/mL 2-nitrophenyl β-D-galactopyranoside) to 20 μL cell lysates. The plates were then incubated for 2 h at 37°C prior to measuring their absorbance at 415 nm using an MTP-310 microplate reader (Corona Electric, Ibaraki, Japan).

The β-gal activity of the cells treated with SBO or SSO was expressed as a percentage of the activity of the DMSO vehicle–treated cells (mean ± standard deviation [SD]). The two groups were then compared using Student’s *t*-test, with differences considered significant at *p* < 0.05.

### Oil red O staining for adipose tissue in medaka

Medaka larvae were sacrificed by immersion into ice chilled water, fixed with 4% paraformaldehyde solution in PBS and rinsed with 60% isopropyl alcohol several times. Subsequently, they were stained with filtered 0.2% Oil red O solution in 60% isopropyl alcohol with gentle rocking at room temperature for 30 min and rinsed with PBS several times. The specimens were observed under a Leica M205FA stereo light/fluorescent microscope with a Leica DFC310 FX camera (Leica, Wetzlar, Germany).

### Preparation of diets for feeding tests

SSO for feeding tests was kindly provided by Daisho Co., Ltd. (Osaka, Japan). Soybean oil (SBO, Riken Nosan-Kako Co., Ltd., Saga, Japan) and rosiglitazone (Cayman Chemical, MI, USA) were obtained commercially. The control diet was prepared by mixing the ingredients listed in Table 1. Rosiglitazone was added to the control diet in a ratio of 50 μg/g, and SSO- and SBO-supplements were added to the control diet in a ratio of 50 or 100 mg/g. All diets were cold extruded, oven-dried overnight at 35°C, and then ground into fine particles. Ground diets were sieved through a 100-nm mesh to obtain micro-particulate diets, which were then packed in sealed tubes with silica gel (as desiccant) and stored at –20°C until use.

**Table 1:** Composition of the control diet.

Ingredients	g/kg diet (dry matter basis)
Fish flesh, raw ^a^	250
Shrimp flesh, raw	200
Polypeptone, casein hydrolysate	150
Egg white powder	140
Dextrin hydrate	30
Carboxymethylcellulose Na salt	20
Cod liver oil	20
Soy lecithin	20
Vitamin premix ^b^	20
Mineral premix ^c^	88
Amino acid premix ^d^	55
Trace mineral solution ^e^	(20 mL)
Total	1000

^a^ A mixture of fresh cod and salmon at a 2:1 ratio

^b^ This premix supplied the following vitamins (mg/kg diet): vitamin A (ca. 25,000 IU), vitamin D_3_ (ca. 2500 IU), DL-α-tocopherol acetate (800), menadione sodium bisulfite (80), thiamine HCl (100), riboflavin (400), nicotinic acid (1480), calcium pantothenate (1000), pyridoxine HCl (100), folic acid (30), cyanocobalamin (0.2), d-biotin (10), choline chloride (10,000), myo-inositol (4000), ascorbic acid (1,000), ascorbate polyphosphate (1000), and butylated hydroxytoluene (BHT; 10). Dextrin was added to a total of 20 g. Vitamins A and D_3_ were supplied in cod liver oil, whereas tocopherol and BHT were dissolved in cod liver oil before being mixed with the other ingredients.

^c^ This premix supplied the following minerals (g/kg diet): NaCl (25), KCl (10), MgSO_4_∙7H_2_O (15), CaHPO_4_∙2H_2_O (18), and KH_2_PO_4_ (20).

^d^ This premix supplied the following amino acids (g/kg diet): DL-methionine (6), L-arginine HCl (5), L-histidine (2), L-lysine HCl (6), Glycine (10), L-threonine (1), DL-α-alanine (10), betaine (5), and taurine (10).

^e^ This solution supplied the following trace minerals (mg/kg diet): KI (8), MnSO_4_∙5H_2_O (250), ZnSO_4_∙7H_2_O (600), Na_2_SeO_3_ (4), CoCl_3_∙6H_2_O (10), CuSO_4_∙5H_2_O (50), FeSO_4_∙7H_2_O (1500), citric acid (5000), and erythrosine (50).

### Quantitative *in vivo* estimation of the adipose tissue volumes using Nile red and feeding tests in medaka larvae

Medaka larvae were collected by the synchronized hatching method, as described by Wakamatsu [33]. In brief, collected eggs from female medaka were incubated at 28°C in the dark with rocking until 7–8 days after fertilization. Once a few larvae hatched, the embryos were put under a light without rocking. The larvae (*n* = 10–20) were transferred into 1-L tanks and fed with either the control diet, rosiglitazone-containing diet (50 μg/g), SSO-supplemented diet (50 mg/g), or SBO-supplemented diet (50 mg/g) twice a day (each 10 mg/tank) for 15 days.

After 15 days of feeding the larvae different diets, the lipophilic stain, Nile red (Sigma-Aldrich, MO, USA) was added to the water at a final concentration of 0.5 μg/mL, and the larvae were placed in the dark for 30 min. Then, they were rinsed with fresh water several times, anesthetized with tricaine methanesulfonate (Wako, Osaka, Japan), and observed under a Leica M205FA stereo light/fluorescent microscope with a Leica DFC310 FX camera and a green fluorescent protein (GFP) filter. The dimensions of the stained area and standard length of the fish, on the same lateral side of the specimens, were measured using ImageJ 1.43r (National Institutes of Health, MD, USA). The Nile red index (stained area/standard length) was calculated, and the values were expressed as the mean ± standard error of the mean (SEM). Statistical analyses were performed using Welch’s *t*-test, Student's *t*-test, Tukey–Kramer’s test, or Williams's test for multiple comparisons.

### Comparative expression analysis of adipogenesis-related genes with quantitative RT-PCR

Ten to twelve larvae collected by the synchronized hatch method were transferred into each of three 1 L tanks for each feeding test, and the fish of each group were fed twice a day for 15 days (each 10 mg/tank). The larvae were collected from each tank and were sacrificed by immersion into ice chilled water. Total RNA was extracted in a lump from every tank with TRizol (Invitrogen, MA, USA). Complementary DNA (cDNA) was prepared from the resulting RNA by using SuperScript III First-Strand Synthesis SuperMix for quantitative reverse transcription-polymerase chain reaction (qRT-PCR; Invitrogen, CA, USA). PCR was performed with the cDNA as a template and each primer set for adipogenesis-related genes and a control gene for normalization, as listed in Table 2, by using Power SYBR Green PCR Mastermix (Applied Biosystems, CA, USA).

**Table 2:** Primer sets for quantitative RT-PCR.

Gene	Gene ID	primer sequences
*β-actin*	S74868.1	5’-GAGCGTGGCTACTCCTTCAC-3’
	(NCBI ID)	5’-AGCACAGTGTTGGCGTACAG-3’
*PPARγ*	NM_001164876	5’-AAGACCACGGAGATCAAGTTCAGG-3’
	(NCBI ID)	5’-ATCTCTCGCTCCAGAGTTGAGGTCT-3’
*ACVR1C*	ENSORLG00000016705	5’-GGAGGCGGAGATTTACCAGACTATC-3’
	(Ensembl ID)	5’-CTTCCACTGAGACGGTGTACCTGTT-3’
*adiponectin*	ENSORLG00000005474	5’-GCAATCCCTGGGGTCTACTTCTTT-3’
	(Ensembl ID)	5’-AACCTCGTCAGAACGCTTTAGGTG-3’
*aP2*	ENSORLG00000008282	5’-AAGACCACGGAGATCAAGTTCAGG-3’
	(Ensembl ID)	5’-ATCTCTCGCTCCAGAGTTGAGGTCT-3’

Real-time detection and analysis of PCR products were performed using the ABI PRISM 7700 Sequence Detection System (Applied Biosystems, CA, USA). The expression levels of these genes were normalized by the expression levels of *β-actin*, and then, the relative values to the expression levels in control-fed medaka were calculated. The values were expressed as the mean ± SEM of three or six independent experiments. Statistical analyses were performed using the Student’s *t*-test or Tukey–Kramer’s test for multiple comparisons.

### Quantitative image analysis of the visceral adipose tissues in the medaka larvae

After the feeding tests, the medaka larvae were collected, sacrificed by immersion into ice chilled water, and fixed with 4% paraformaldehyde solution in PBS. According to manufacturer’s instruction, the medaka larvae were embedded in plastic resins (Technovit 8100; Heraeus Kulzer, Hanau, Germany). The tissue blocks were cut into serial thin sections (thickness, 5 μm) with a rotary microtome LEICA RM2235 (Leica Microsystems, Wetzlar, Germany) and placed onto glass slides. The sections were stained with hematoxylin and eosin (H&E), and then, the number and area of the adipocytes and area of the tissues on the randomly selected sections (three sections per fish) were measured using ImageJ 1.43r. Data were expressed in terms of the median and interquartile ranges. Statistical analyses were performed using the Mann–Whitney *U* test or Steel–Dwass’ test for multiple comparisons.

## Results

### Quantitative *in vivo* estimation of the adipose tissue volumes with Nile red staining in the medaka

**Fig 1:**
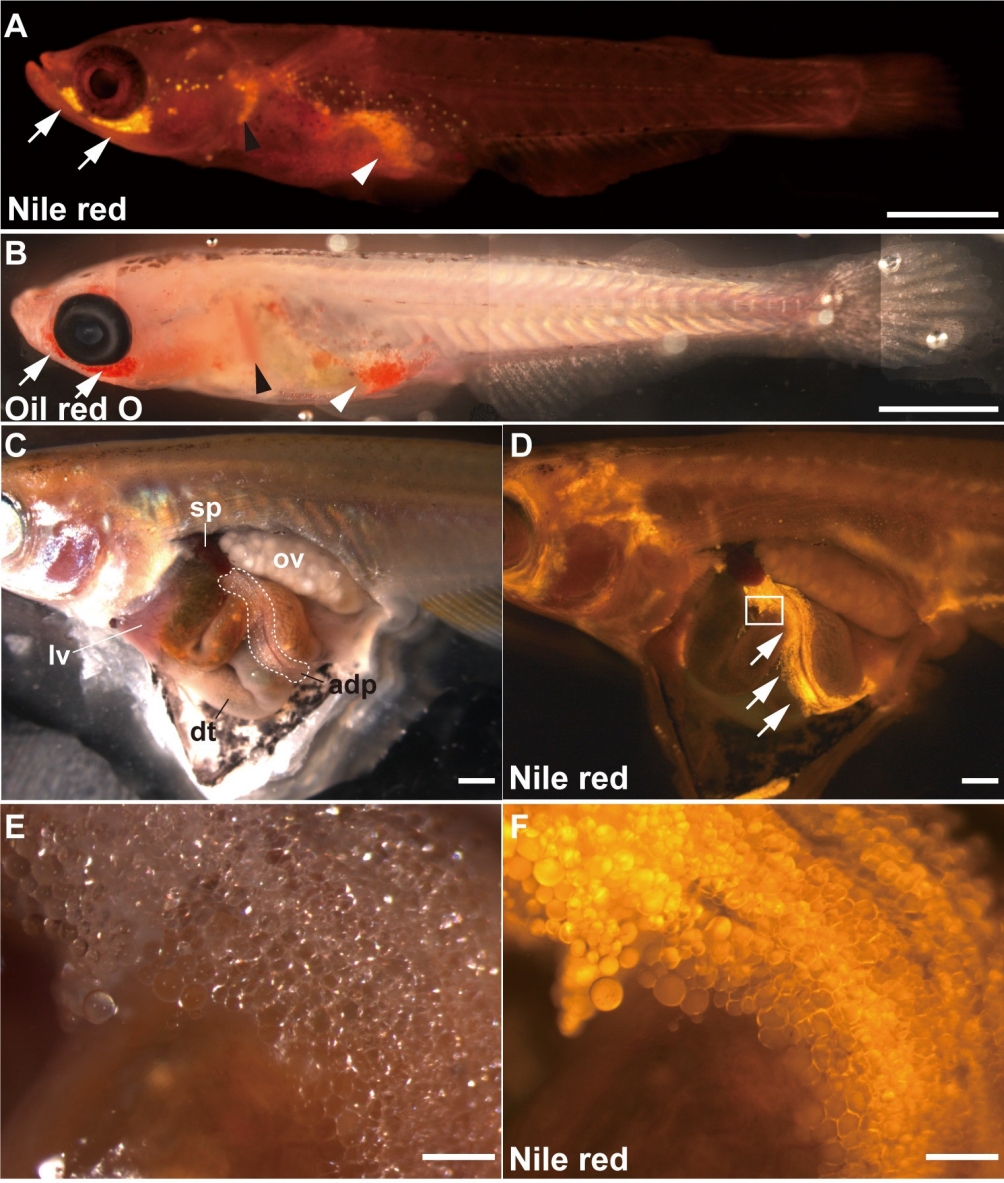
Nile red can stain adipose tissues in living medaka. (A) Medaka larva at 15 days post-hatch (dph) stained with Nile red. (B) Medaka larva at 15 dph stained with Oil red O. Nile red stained the same parts of the larvae as Oil red O. These included under the eyes (arrows), at the base of the pectoral fin (black arrowhead), along the dorsal wall of the abdominal cavity, and around the sides of the anus (compare A and B, white arrowhead). Scale bar = 1 mm. (C and D) Adult female medaka was stained with Nile red. (C) Stereomicroscopic image. (D) Fluorescent stereomicroscopic image. The dashed circle area indicates adipose tissues in panel C. adp, adipose tissue; dt, digestive tract; lv, liver; ov, ovary; sp, spleen. Fluorescence signals were observed in visceral adipose tissues (D, arrows). Scale bar = 1 mm. (E and F) Magnified images of the boxed area in panel D. (E) Stereomicroscopic image. (F) Fluorescent stereomicroscopic image. Fluorescence signals were apparent in many adipocytes. Scale bar = 200 μm.

To study the effects of dietary soy sauce oil (SSO) on adipogenesis *in vivo*, we designed a technique for quantitatively estimating the adipose tissue volumes by Nile red staining in medaka. Following uptake by living organisms, the Nile red is distributed into adipose tissue, where it binds to lipids. Therefore, using this stain enabled us to visualize the oil droplet–rich cells in the adipose tissue under 588 nm excitation. Nile red has previously been used to stain adipose tissue or oil droplets in cells in several model organisms, including zebrafish, nematodes, and yeast [34–36]. To confirm that Nile red could be used to stain adipose tissue in medaka, 15 dph (days post-hatch) medaka larvae were stained with Nile red or with Oil red O (which is traditionally used to stain adipose tissue). We found that Nile red stained the same parts of the larvae as Oil red O, i.e., under the eyes, at the base of the pectoral fin, along the dorsal wall of the abdominal cavity, and around the sides of the anus (Fig 1A and 1B, arrows, black arrowhead, and white arrowhead). Furthermore, in adult female medaka, strong Nile red signals were observed in a tissue that was subsequently identified as adipose tissue based on its anatomical features, i.e., its location and the mass of oil droplet-rich cells it contained (Fig 1C–1F). These results suggest that Nile red can stain adipose tissue in living medaka.

On the basis of physiological roles and anatomical features, adipose tissues are classified as white adipose tissue (WAT) and brown adipose tissue (BAT) [1]. Studies have shown that WAT is involved in storing energy in the form of triglycerides and in metabolism, whereas BAT is involved in heat production [2]. Consequently, feeding conditions particularly affect the WAT volumes in the body.

**Fig 2:**
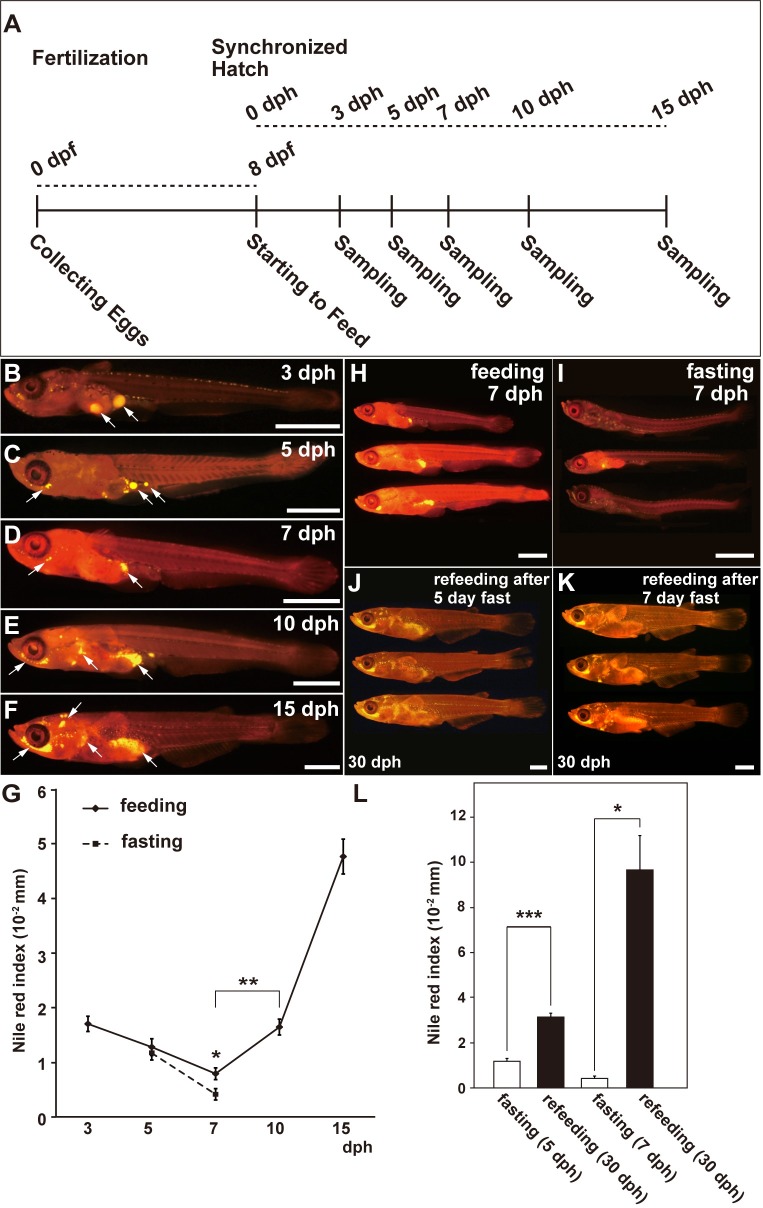
Quantitative *in vivo* estimation of the adipose tissue volumes by Nile red staining in the medaka larvae. (A) Schedule for the feeding tests. Here, dpf indicates day post-fertilization, and dph indicates day post-hatch. (B–F) Fluorescent images of the medaka larvae stained with Nile red at selected stages (lateral view). Fluorescent signals were observed in the yolk droplets (B, C, arrows at the abdominal cavity) and in the adipose tissues (C, arrow under the eye, and D–F, arrows). (G) Comparison of changes in Nile red indexes (stained area/standard length) between the feeding and fasting groups. (H) The medaka larvae in the feeding group at 7 dph. (I) The medaka larvae in the fasting group at 7 dph. (J) The medaka larvae at 30 dph in the refeeding group after 5 days of fasting. (K) The medaka larvae at 30 dph in the refeeding group after 7 days of fasting. (L) Comparison of the changes in Nile red indexes (stained area/standard length) between the fasting and refeeding groups. Data are expressed as mean ± SEM. Statistical analyses were performed using Student’s *t*-test (G and L) and Williams’ test for multiple comparisons (G). *p < 0.05; **p < 0.01; ***p < 0.001. Scale bar = 1 mm.

To develop an *in vivo* technique to estimate adipose tissue volumes quantitatively using Nile red, we performed feeding tests using medaka larvae (Fig 2A). We found that the Nile red index (stained area/standard length) decreased from 3 to 7 dph and then increased from 7 to 15 dph (Fig 2B–2F, arrows, and G). At 0–3 dph, the medaka larvae still contained a yolk droplet in the abdomen, which was stained by Nile red (data not shown). The size of this yolk droplet gradually decreased from 3 to 7 dph (Fig 2B–2D, arrows, and G). The Nile red signals that refer to adipose tissues first appeared at 7 dph and then increased until 15 dph (Fig 2D–2F, arrows, and G). In the period of increase (7–15 dph), Nile red index at 10 dph was significantly higher compared to that at 7 dph (Fig 2G, *p* < 0.01, William’s test). Additionally, at 5 dph, the Nile red indexes corresponding to fasting and feeding conditions were comparable; however, at 7 dph, the Nile red indexes were significantly lower under fasting conditions than those under feeding conditions (Fig 2G–2I, comparing 7 dph larvae under the feeding condition [*n* = 42] with 7 dph larvae under the fasting condition [*n* = 26], *p* = 0.021, Student’s *t*-test). Importantly, the Nile red indexes significantly increased upon refeeding following 5 and 7 days of fasting (Fig 2J–2L, 5 dph larvae under fasting condition [*n* = 38] versus 30 dph larvae under refeeding condition [*n* = 45], *p* = 9.68 × 10^–14^; 7 dph larvae under fasting condition [*n* = 26] versus 30 dph larvae under refeeding condition [*n* = 3], *p* = 0.025, Student’s *t*-test). These results suggest that the Nile red index can be used to quantitatively assess changes in the WAT volumes and that the optimal duration for a feeding test is at least 10 days.

**Fig 3:**
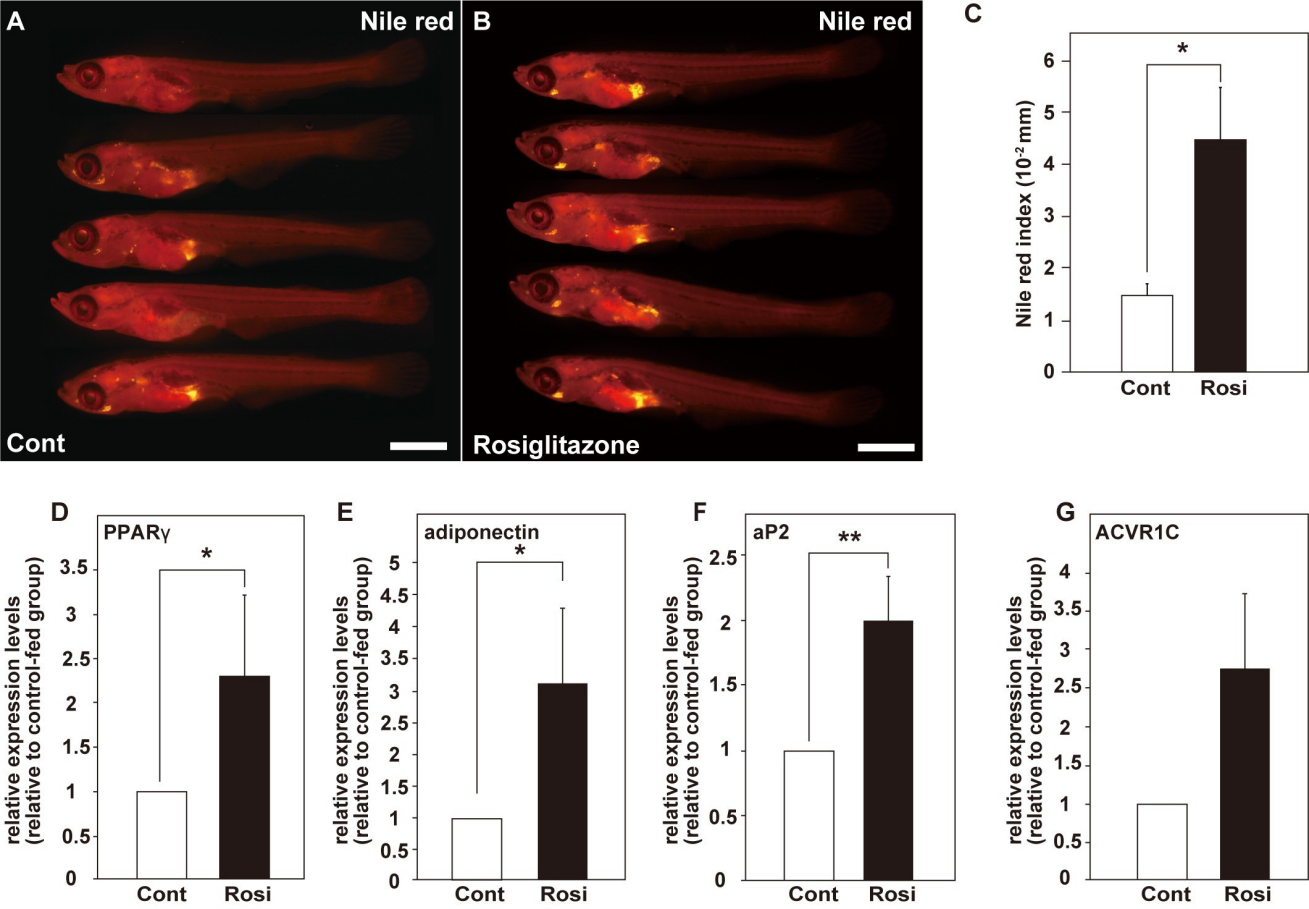
Oral intake of rosiglitazone promotes adipogenesis in the medaka larvae. (A and B) The medaka larvae were stained with Nile red after the feeding test. Scale bar = 1 mm. (A) The control diet-fed medaka larvae. (B) The 50 μg/g rosiglitazone-administered medaka larvae. (C) Comparison of Nile red indexes (stained area/standard length) between the control diet-fed and rosiglitazone-administered groups. (D–G) The expression levels of adipogenesis-related marker genes were compared by qRT-PCR in the control- or rosiglitazone-administered medaka groups. (D) *PPARγ*. (E) *adiponectin*. (F) *aP2*. (G) *ACVR1C*. The values were expressed as the mean ± SEM of six independent experiments. Statistical analyses were performed using Welch’s *t*-test (C) or Student’s *t*-test (D–G). *p < 0.05; **p < 0.01; ***p < 0.001; ns indicates not significant.

To verify whether the quantitative estimation method can detect changes in the adipose tissue volumes caused by various dietary treatments, we performed a feeding test with rosiglitazone, which is known to be a potent PPARγ agonist [37]. During the feeding test, the oral intake of rosiglitazone did not affect viability and growth in the medaka larvae (Fig 3A and 3B). Expectedly, in the rosiglitazone-administered medaka larvae, the Nile red index significantly increased compared with that in control-fed medaka larvae (Fig 3C, control [*n* = 12] versus rosiglitazone [*n* = 13], *p* = 0.018, Welch’s *t*-test). Moreover, to further confirm that the oral intake of rosiglitazone promotes preadipocyte differentiation, we performed a comparative expression analysis of the adipogenesis-related genes such as *PPARγ*, *ACVR1C*, *adiponectin*, and *aP2* between the control group and rosiglitazone-administered group by qRT-PCR. In the rosiglitazone-administered medaka larvae, the expression levels of the adipogenesis marker genes such as *PPARγ* (Fig 3D, *p* = 0.0396, *n* = 6, Student’s *t*-test), *adiponectin* (Fig 3E, *p* = 0.0339, *n* = 6), and *aP2* (Fig 3F, *p* = 0.0024, *n* = 6) significantly increased; *ACVR1C* also tended to increase to levels higher than that in the control group (Fig 3G, *p* = 0.0759, *n* = 6), indicating that the oral intake of rosiglitazone effectively promotes adipogenesis in the medaka larvae. Taken together, the promotive effects of PPARγ agonist on the adipose tissue volumes were detected by our quantitative method using the medaka. Thus, the feasibility of using this method to estimate the effects of dietary treatments on adipogenesis *in vivo* was validated.

### Soy sauce oil feeding promotes adipogenesis through PPARγ activation in medaka

**Fig 4:**
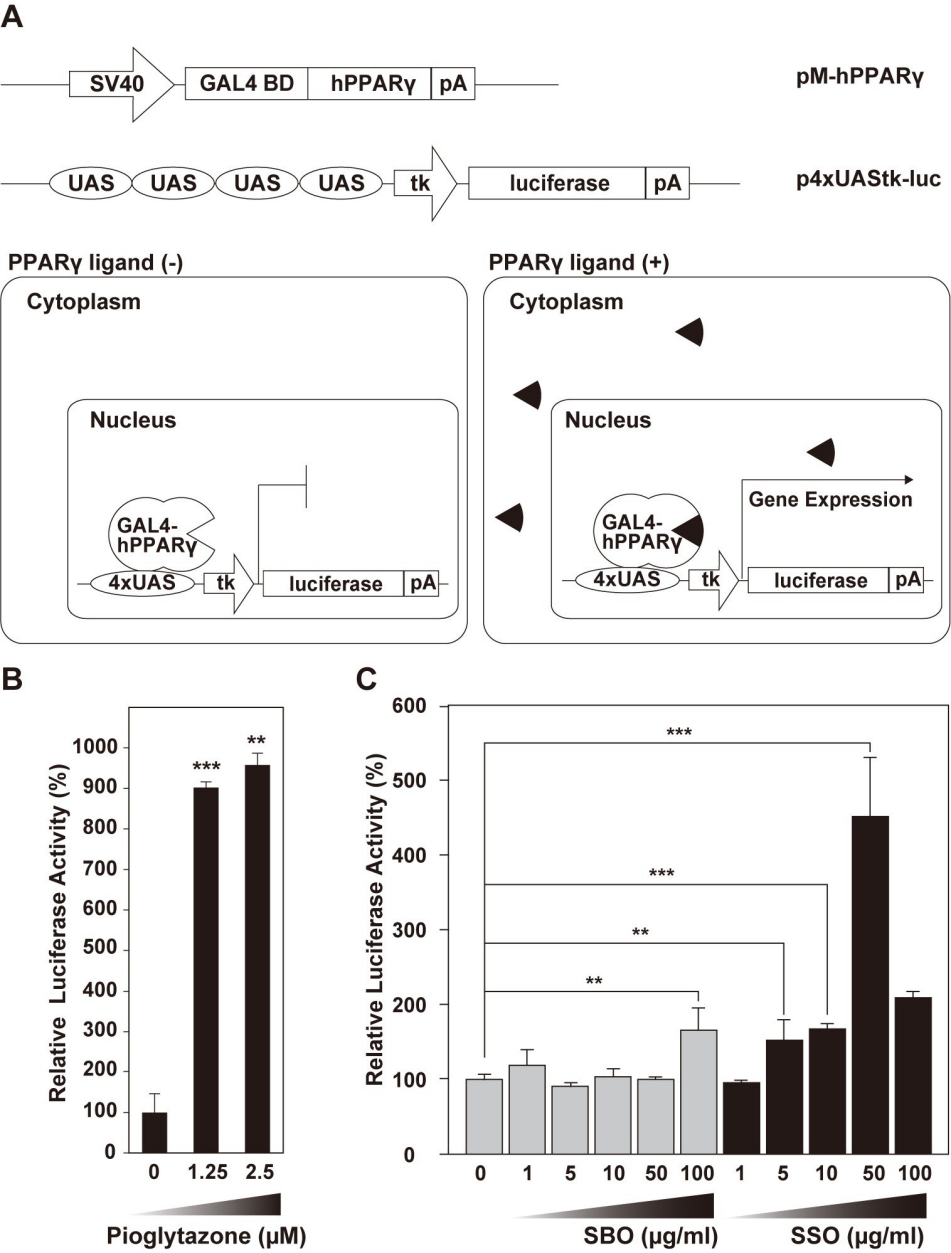
*In vitro* PPARγ-agonistic activity of soy sauce oil. (A) Schematic representation of the luciferase assays of PPARγ-agonistic activity. SV40, simian virus 40; GAL4BD, GAL4 DNA-binding domain; pA, poly A tail; UAS, upstream-activating sequence; tk, thymidine kinase. Closed sectors indicate PPARγ ligands that form GAL4–hPPARγ–ligand complexes in the nucleus, leading to specific induction of the reporter gene luciferase. (B) Relative luciferase activity in PPARγ agonist, pioglitazone-treated cells (positive control). (C) Relative luciferase activity in soybean oil (SBO)-treated and soy sauce oil (SSO)-treated cells. The values were expressed as mean ± SD. The statistical analyses were performed using the Student’s *t*-test. *p < 0.05; **p < 0.01; ***p < 0.001.

To confirm that the materials collected for use in this study had PPARγ agonistic activity, we performed a cell-based reporter assay using the *GAL4*-UAS system (Fig 4A). The GAL4 DNA-binding domain (GAL4BD) directly binds to the upstream-activating sequence (UAS) and can activate the expression of downstream genes. The gene coding for a fusion protein containing GAL4BD and human PPARγ is driven by a strong, ubiquitous simian virus 40 (SV40) promoter. The luciferase reporter gene is driven by the UAS and the thymidine kinase promoter. When ligands exist in the media, the PPARγ-ligand complexes in the nucleus induce the expression of the luciferase reporter gene. Therefore, luciferase activity is directly related to the concentration of ligands. When the cells were treated with pioglitazone, as a positive control [37], high levels of luciferase activity were detected in a concentration-dependent manner, indicating that the assay system worked normally (Fig 4B, comparison of 0-μM treatment with 1.25-μM treatment: *p* = 0.0007, *n* = 4; 2.5-μM treatment: *p* = 0.0026, *n* = 4).

Soy sauce oil (SSO) is generated from soybeans during fermentation. The relative effects of SSO and soybean oil (SBO) treatments could reflect the existence of active compounds that are produced during fermentation. Thus, PPARγ-agonistic activities of SBO and SSO were investigated using cellular luciferase assays after treatments with SBO and SSO (Fig 4C). In both groups, compared to the control group, the luciferase activity gradually increased in a concentration-dependent manner, although in SSO-treated cells at a concentration of 100 μg/mL, activity measurement was disturbed because of detachment of cells. Moreover, in SSO-treated cells at a concentration of 5–50 μg/mL, the luciferase activity significantly increased compared to the control group (comparison of 0-μg/mL treatments with 5-μg/mL SSO, *p* = 0.0013 [*n* = 6]; 10 μg/mL SSO, *p* = 1.16 × 10^−12^ [*n* = 6]; 50 μg/mL SSO, *p* = 1.82 × 10^−8^ [*n* = 6]). In contrast, SBO treatments at 100 μg/mL significantly increased luciferase activity compared with the control group (*p* = 0.0003, *n* = 6), suggesting that the minimum concentration of SSO that can significantly increase luciferase activity is lower than that of SBO. Taken together, these results indicate that both SBO and SSO have PPARγ-agonistic activity and that the activity of SSO is higher than that of SBO.

**Fig 5:**
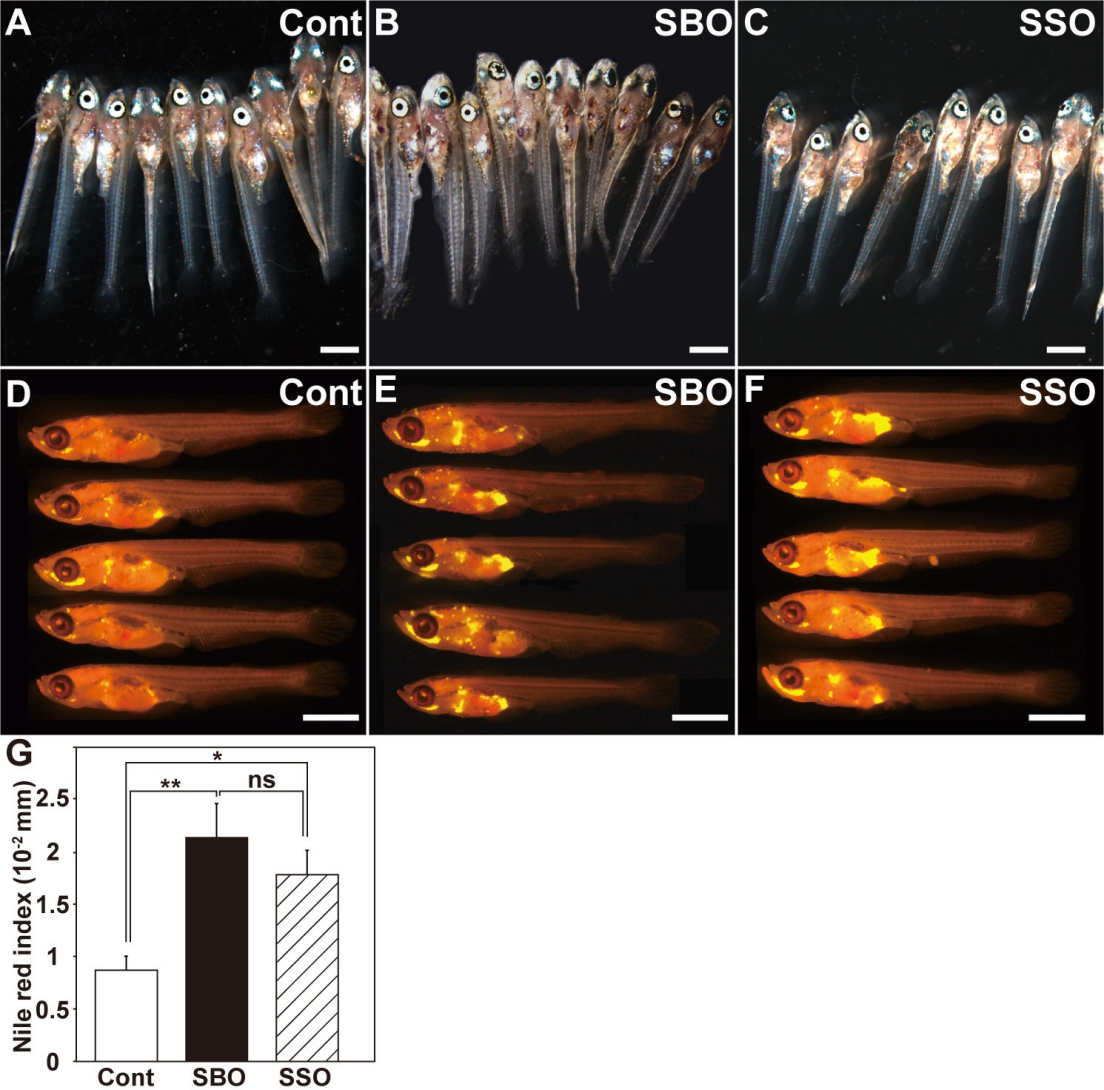
Soy sauce oil feeding increases the adipose tissue volumes in the medaka larvae. (A–C) The medaka larvae were fed with the control diet (A), the SBO-supplemented diet (B), and the SSO-supplemented diet (C) for 15 days. No significant differences were observed between the feeding groups with respect to morphology. (D–F) The medaka larvae were stained with Nile red after feeding tests with the control diet (D), the SBO-supplemented diet (E), and the SSO-supplemented diet (F) for 15 days. Scale bar = 1 μm (A–F). (G) Comparison of Nile red indexes (stained area/standard length) among the control-, SSO-, and SBO-fed groups. Data are expressed as mean ± SEM. Statistical analyses were performed using the Tukey–Kramer’s test for multiple comparisons; *p < 0.05; **p < 0.01; ***p < 0.001; ns indicates not significant.

To study the effects of dietary SSO treatment on adipogenesis in medaka larvae, we performed the feeding tests with the control diet, SSO-supplemented diet, or SBO-supplemented diet. There was no difference between the SSO-fed, SBO-fed, and control groups at 15 dph with respect to body length, weight, body shape, or the amount of food intake (Fig 5A–5C, and data not shown). In both SBO- and SSO-fed medaka, the Nile red index significantly increased compared to that in the control group (cont [*n* = 17] versus SSO [*n* = 11], *p* = 0.033; cont [*n* = 17] versus SBO [*n* = 18], *p* = 0.006, Tukey–Kramer’s test); however, the Nile red index in SSO-fed medaka was comparable to that in SBO-fed medaka (Fig 5D–5F and 5G, SBO [*n* = 18] versus SSO [*n* = 11], *p* = 0.564, Tukey–Kramer’s test). These results suggest that both SBO and SSO feeding increase adipose tissue volumes *in vivo*.

**Fig 6:**
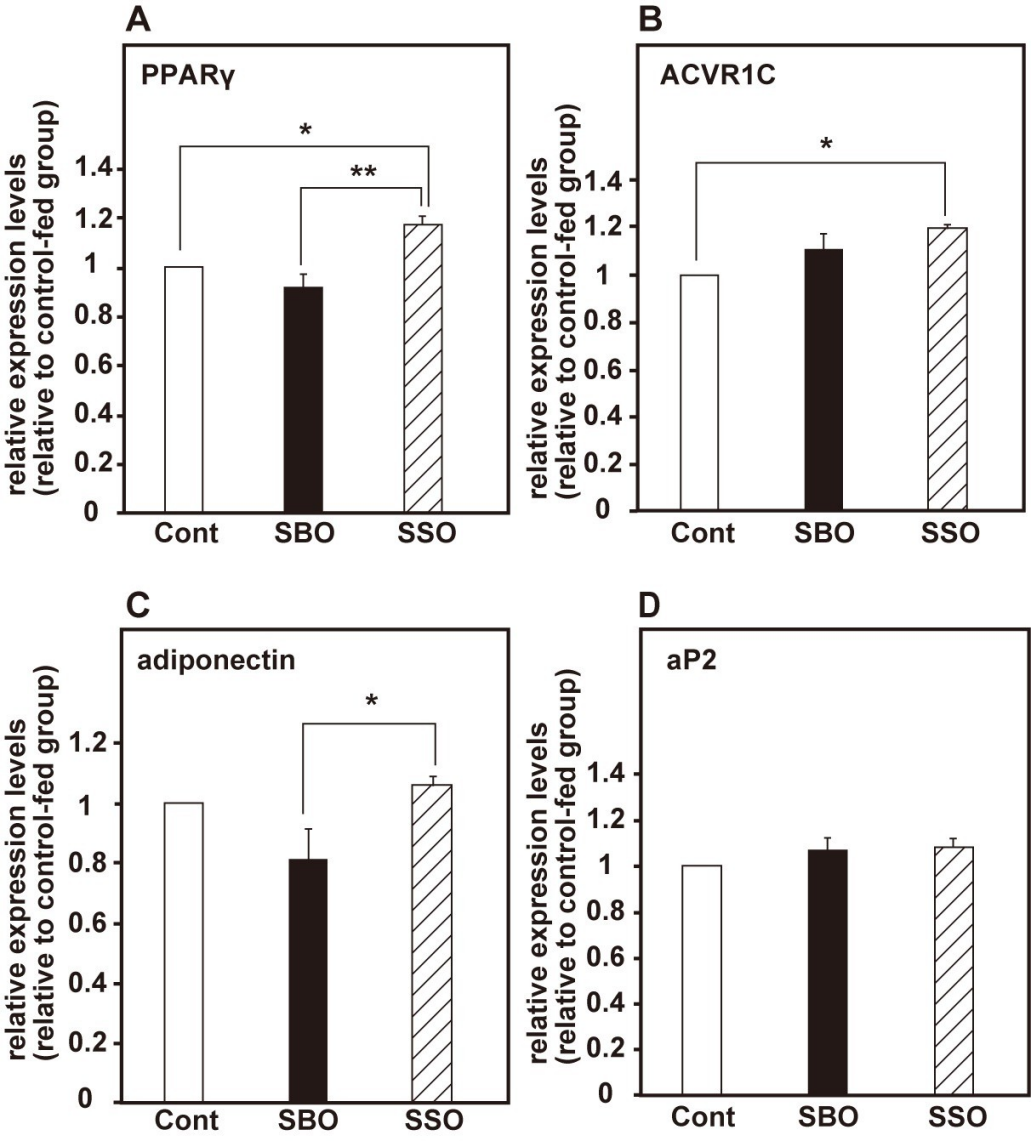
Soy sauce oil feeding increases the expression levels of adipogenesis-related genes *in vivo*. The expression levels of adipogenesis-related marker genes such as *PPARγ* (A), *ACVR1C* (B), *adiponectin* (C), and *aP2* (D) was compared by qRT-PCR in the control group, the SBO-fed group, and the SSO-fed group. The values were expressed as the mean ± SEM of three independent experiments. Statistical analyses were performed using the Tukey–Kramer’s test for multiple comparisons. *p < 0.05; **p < 0.01; ***p < 0.001.

To study whether SBO and SSO feeding promotes preadipocyte differentiation, we performed comparative expression analysis of adipogenesis-related genes by qRT-PCR. We found that the expression levels of *PPARγ* in SSO-fed medaka significantly increased compared to control-fed and SBO-fed medaka (Fig 6A; cont versus SBO, *p* = 0.230; cont versus SSO, *p* = 0.019; SBO versus SSO, *p* = 0.006, *n* = 3). The expression level of *ACVR1C* significantly increased in SSO-fed medaka compared to control-fed medaka (Fig 6B, *p* = 0.022, *n* = 3). Moreover, the expression level of *adiponectin* significantly increased in SSO-fed medaka compared to SBO-fed medaka (Fig 6C, *p* = 0.032, *n* = 3), although the expression of *aP2* did not change among the three groups (Fig 6D). These results suggest that adipogenesis is promoted in, especially, SSO-fed medaka rather than in SBO-fed medaka.

**Fig 7:**
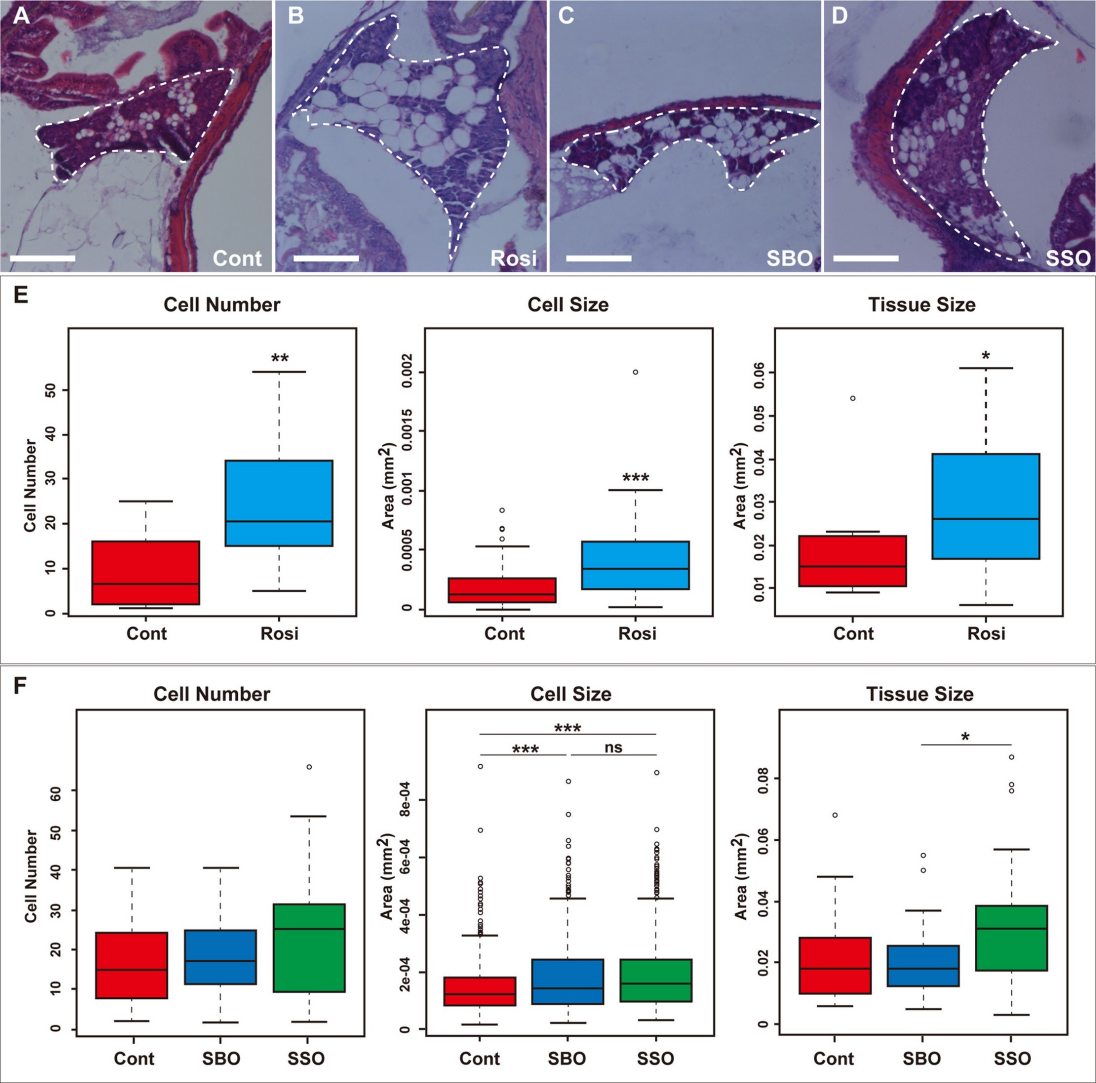
Quantitative image analysis of the visceral adipose tissues in the dietary treated medaka larvae. (A–D) Hematoxylin and eosin stained sections of the visceral adipose tissues prepared from control group (A), rosiglitazone (Rosi)-administered group (B), soybean oil (SBO)-fed group (C), and soy sauce oil (SSO)-fed group (D). Scale bar = 0.1 μm. Regions depicted by dashed circles indicate the visceral adipose tissues. (E) Quantitative image analysis of the visceral adipose tissues on HE-stained sections prepared from the rosiglitazone-administered medaka larvae. The cell number, cell area, and tissue area on randomly selected sections (three sections per fish) were measured. Data were expressed in terms of the median and interquartile range. The box plots of the data sets were drawn with outliers (open circles). Statistical analyses were performed using the Mann–Whitney *U* test. *p < 0.05; **p < 0.01; ***p < 0.001. (F) Quantitative image analysis of the visceral adipose tissues on HE-stained sections prepared from the control, SBO-fed, and SSO-fed groups. The cell number, cell area, and tissue area on randomly selected sections (three sections per fish) were measured. Data were expressed in terms of the median and interquartile range. The box plots of the data sets were drawn with outliers (open circles). Statistical analyses were performed using the Steel–Dwass’ test for multiple comparisons. *p < 0.05; **p < 0.01; ***p < 0.001; ns indicates not significant.

Next, to further investigate the effects of dietary SSO on adipogenesis at the tissue level, we performed a quantitative image analysis of the visceral adipose tissues derived from the medaka larvae after the feeding tests (Fig 7A–7D). We found that in rosiglitazone-administered group (*n* = 6), the number and area of the adipocytes and area of the visceral adipose tissues significantly increased compared with those in the control group (*n* = 4) (Fig 7E, cell number: *p* = 0.0025; cell size: *p* < 2.2 × 10^−16^; tissue size: *p* = 0.0416, Mann–Whitney *U* test), indicating that intake of rosiglitazone promotes expansion of the visceral adipose tissues. Interestingly, similar to these results, in the SSO-fed medaka larvae (*n* = 9), a significant increase in the area of the adipocytes compared with that in the control group (*n* = 10) was observed, although the rate of increase was the same as in the SBO-fed group (*n* = 8), and the area of the tissues also significantly increased compared with at least the SBO-fed group (Fig 7F, cell size: cont versus SSO, *p* = 3.35 × 10^−11^; SBO versus SSO, *p* = 0.424; tissue size: SBO versus SSO, *p* = 0.0445, Steel–Dwass’ test). Additionally, in the SSO-fed group, the number of the adipocytes tended to be higher than that in the control group and SBO-fed group, although the differences were not significant (Fig 7F, cell number). In contrast, in the SBO-fed group, a significant increase in the area of the adipocytes was observed compared with that in the control group, whereas the number of the adipocytes and area of the tissues were comparable with those in the control group (Fig 7F, cell size: cont versus SBO, *p* = 4.67 × 10^−6^, Steel–Dwass’ test). These results suggest that SSO feeding results in higher PPARγ agonistic activities *in vivo* than SBO feeding.

## Discussion

In this study, we examined the effects of dietary SSO on adipogenesis by using a medaka model as an *in vivo* experimental system and have demonstrated that SSO feeding increases the adipose tissue volumes through adipogenesis promotion by the PPARγ-agonistic activity in medaka. We found four lines of evidence to support our conclusion: (i) SSO and SBO have PPARγ-agonistic activity, with SSO having higher activity than SBO (Fig 4); (ii) SBO- and SSO-supplemented diets increased the adipose tissue volumes in medaka (Fig 5); (iii) the expression levels of adipogenesis-related genes increased more in SSO-fed medaka compared to SBO-fed medaka (Fig 6); and (iv) the SSO-fed medaka exhibited parameter changes in the visceral adipose tissues similar to those seen in the case of the rosiglitazone-administered medaka, rather than in the case of the SBO-fed group (Fig 7). Although SSO feeding increased the expression level of *PPARγ in vivo*, we did not detect remarkable expression of the PPARγ-target genes such as *aP2* and *adiponectin* in SSO-fed medaka (Fig 6). In contrast, in the rosiglitazone-administered medaka, a significant increase was detected in the expression levels of *PPARγ* and *aP2* (Fig 3). Because of the difficulty in isolating adipose tissues from medaka larvae, for qRT-PCR, total RNA was extracted from the entire body of the larvae. We found that *aP2* was expressed in the gonads, besides adipose tissue (data not shown). This probably interrupted the detection of slight differences in the expression level of *aP2* among the three groups. Therefore, more marker genes that act downstream of *PPARγ* and can indicate a change in the gene expression, even if it is tested with whole medaka larvae, need to be identified. Additionally, in spite of the slight increase in the number and size of the adipocytes in the SSO-fed group compared with those in the SBO-fed group (Fig 7), quantitative estimation using Nile red staining was not sufficiently sensitive to detect the differences between the visceral adipose tissues in the SBO- and SSO-fed groups (Fig 5). It is likely that a more effective way to increase the sensitivity is extending the period of the feeding test; however, the optimization of experimental conditions is necessary to strike a balance among the ease, cost, and sensitivity.

Our results indicate the novel effects of fermented foods on adipogenesis, although the components of SSO that promote adipogenesis *in vivo* have not been identified. Soybean fermentation likely produces compounds that play important roles in adipogenesis in medaka. Among these, genistein is abundant in SSO and is generated from isoflavone glycoside [38]. Additionally, genistein reportedly acts as a PPARγ ligand [21] and induces adipogenesis in rat synovial fibroblasts [39, 40] and in male mice fed a low-fat diet [41]. In contrast, genistein suppresses adipogenesis and increases lipolysis in 3T3-L1 cells [42–44]. These effects reportedly depend on genistein concentrations [21]; that is, high-concentration genistein treatment inhibits adipogenesis, whereas low-concentration treatment promotes adipogenesis *in vivo* and *in vitro*. Therefore, the *in vivo* and *in vitro* effects of genistein on adipogenesis remain controversial. Moreover, free fatty acids are present at higher concentrations in SSO than in SBO [5], and various polyunsaturated acids, including linoleic acid, linolenic acid, and arachidonic acid reportedly act as selective PPARγ ligands [45, 46]. Indeed, SSO treatment exhibits higher PPARγ-agonistic activity *in vitro* and higher adipogenesis promotion *in vivo* than SBO treatment. However, Kawachi and colleagues have shown that when SSO was fractionated with reverse-phase column chromatography, even a fraction that contains no linoleic acid has PPARγ-agonistic activity [24], suggesting that linoleic acid is not a candidate. Therefore, further efforts should be dedicated toward identifying active compounds in SSO.

In this study, we have demonstrated that the medaka is a powerful model to study adipogenesis *in vivo* in terms of basic developmental biology and search for compounds affecting adipogenesis. It is noteworthy that hPPARγ was used in the luciferase assay; however, adipogenesis was promoted in medaka larvae, suggesting that the mechanism of adipogenesis is conserved among vertebrate species. The deduced amino acid sequence of human *PPARγ* (NCBI Gene ID: NP_056953) shows relatively high homology to that of *PPARγ* from medaka (NP_001158384, 60%) and other fish including zebrafish (NP_571542, 64%), puffer fish (NP_001091096, 61%), and rainbow trout (NP_001184141, 67%). Indeed, we previously performed feeding tests with SSO and SBO on Biwa salmon (*Oncorhynchus masou rhodurus*) to study the effects of SSO on adipose tissue and growth. Consistent with our results obtained with medaka, the fat rate was unchanged between SBO and SSO feeding, although SSO feeding slightly affected the flavor of the fish [5]. Notably, Nile red staining method for *in vivo* quantitative estimation of adipose tissue volumes was even more sensitive than the total lipid weight measuring method, because when total lipid was extracted from the same number of medaka larvae used for Nile red staining and then their dry weight was measured, they were below detection limit in some cases. Quantitative image analysis showed that rosiglitazone intake and SSO feeding caused an increase in the number and size of the adipocytes in the visceral adipose tissues, resulting in the tissue expansion. To investigate the responses to dietary SSO treatment in the distinct types of adipose tissues, we performed a quantitative image analysis of the infraorbital subcutaneous adipose tissues of SBO- and SSO-fed medaka larvae (S1 Fig). Surprisingly, unlike in the visceral adipose tissues, in the SBO- and SSO-fed groups, the number of the adipocytes significantly decreased compared with that in the control group, whereas, especially in the SSO-fed group, a significant increase in cell size compared with that in the other group was observed. These results indicate the possibilities that different molecular mechanisms of adipogenesis act in the visceral adipose tissues and subcutaneous adipose tissues with respect to response to PPARγ agonists. We cannot explain these mechanisms in detail, but it is noteworthy that the medaka model allows analysis of the distinct types of adipose tissues, such as subcutaneous and visceral adipose tissues. Some studies for screening of PPARγ agonists using zebrafish have already been reported; however, these studies have not analysed the effects of dietary feeding [34, 47]. In contrast, our feeding test on the medaka model was performed with dietary SSO and SBO under natural conditions, in which physiological factors such as absorption, distribution, metabolism, and excretion were considered. Moreover, medaka has unique advantages over zebrafish: medaka can survive in a broad temperature range, and they have a well-known mechanism of sex differentiation (which should be considered in the study of adipogenesis) [48]. Finally, recent studies have reported that various techniques such as genetics, transgene, and genome editing by CRISPR/Cas system can be applied to medaka [49–52]. Therefore, further *in vivo* studies of adipogenesis in medaka by using these techniques will yield important insights into manipulating quality and quantity of adipose tissues in farmed fish and livestock animals.

## Supporting information

S1 Fig Quantitative image analysis of the infraorbital adipose tissues in the dietary-treated medaka larvae. (A–C) Infraorbital subcutaneous adipose tissues in control group (A), soybean oil (SBO)-fed group (B), and soy sauce oil (SSO)-fed group (C). Scale bar = 0.1 μm. Regions circled by the dashed line indicate parts of the infraorbital subcutaneous adipose tissues. (D) Quantitative image analysis of the infraorbital subcutaneous adipose tissues in these dietary-treated medaka larvae. The number and area of adipocytes, which compose the tissues on both sides of the fish body, were measured (five fish per feeding test group). Data were expressed in terms of the median and interquartile range. The box plots of the data sets were drawn with outliers (open circles). In the SBO-fed group and SSO-fed group, the number of adipocytes significantly decreased compared with that in the control group (cont versus SBO, *p* = 0.0025; cont versus SSO, *p* = 0.00081; SBO versus SSO, *p* = 0.608). In contrast, a significant increase in cell size compared with that in the other group (cont versus SBO, *p* = 0.082; cont versus SSO, *p* = 2.03 × 10^−14^; SBO versus SSO, *p* = 2.61 × 10^−7^) was observed. Statistical analyses were performed using the Steel–Dwass’ test for multiple comparisons. *p < 0.05; **p < 0.01; ***p < 0.001; ns indicates not significant.
(TIF)
