# Supplementary figures and images for: Establishment of a quantitative in vivo method for estimating adipose tissue volumes and the effects of dietary soy sauce oil on adipogenesis in medaka, Oryzias latipes
Source: PLoS One. 2018 Oct 18;13(10):e0205888. doi: 10.1371/journal.pone.0205888 (PMC6193695; doi:10.1371/journal.pone.0205888)

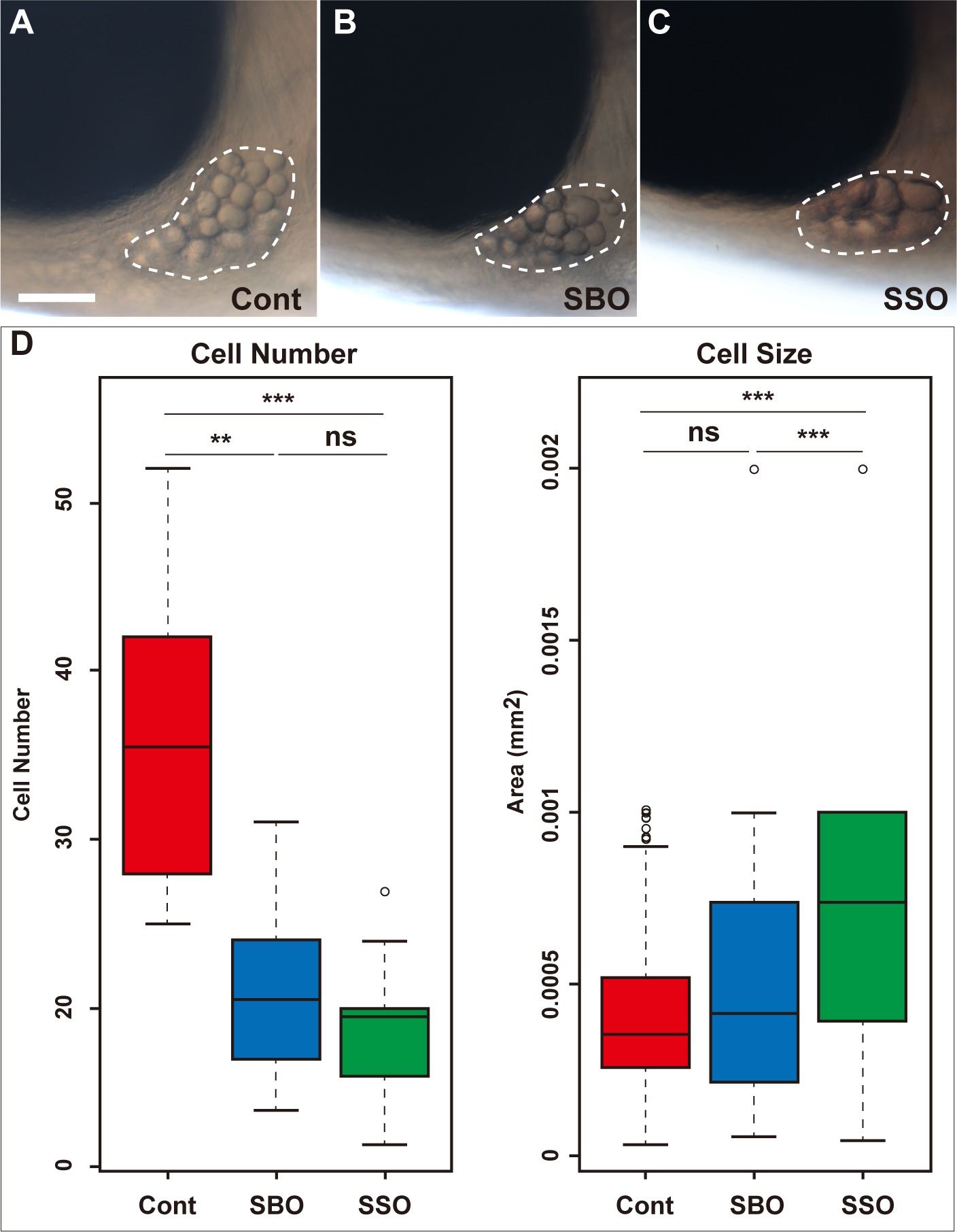

Supplement: S1 Fig — (A–C) Infraorbital subcutaneous adipose tissues in control group (A), soybean oil (SBO)-fed group (B), and soy sauce oil (SSO)-fed group (C). Scale bar = 0.1 μm. Regions circled by the dashed line indicate parts of the infraorbital subcutaneous adipose tissues. (D) Quantitative image analysis of the infraorbital subcutaneous adipose tissues in these dietary-treated medaka larvae. The number and area of adipocytes, which compose the tissues on both sides of the fish body, were measured (five fish per feeding test group). Data were expressed in terms of the median and interquartile range. The box plots of the data sets were drawn with outliers (open circles). In the SBO-fed group and SSO-fed group, the number of adipocytes significantly decreased compared with that in the control group (cont versus SBO, p = 0.0025; cont versus SSO, p = 0.00081; SBO versus SSO, p = 0.608). In contrast, a significant increase in cell size compared with that in the other group (cont versus SBO, p = 0.082; cont versus SSO, p = 2.03 × 10^−14^; SBO versus SSO, p = 2.61 × 10^−7^) was observed. Statistical analyses were performed using the Steel–Dwass’ test for multiple comparisons. *p < 0.05; **p < 0.01; ***p < 0.001; ns indicates not significant. (TIF) [file pone.0205888.s001.tif]
